# Simultaneous quantification of salivary 3-hydroxybutyrate, 3-hydroxyisobutyrate, 3-hydroxy-3-methylbutyrate, and 2-hydroxybutyrate as possible markers of amino acid and fatty acid catabolic pathways by LC–ESI–MS/MS

**DOI:** 10.1186/s40064-015-1304-0

**Published:** 2015-09-15

**Authors:** Teruo Miyazaki, Akira Honda, Tadashi Ikegami, Junichi Iwamoto, Tadakuni Monma, Takeshi Hirayama, Yoshifumi Saito, Kouwa Yamashita, Yasushi Matsuzaki

**Affiliations:** Joint Research Center, Tokyo Medical University Ibaraki Medical Center, Ami, Japan; Division of Gastroenterology and Hepatology, Department of Internal Medicine, Tokyo Medical University Ibaraki Medical Center, 3-20-1, Chuo, Ami, Inashiki, Ibaraki 300-0395 Japan; Laboratory of Analytical Chemistry, Department of Kampo Pharmacy, Yokohama University of Pharmacy, Yokohama, Kanagawa Japan

## Abstract

We have developed a highly sensitive and specific method for quantification of salivary 3-hydroxybutyrate (3HB), 3-hydroxyisobutyrate (3HIB), 3-hydroxy-3-methylbutyrate (3HMB) and 2-hydroxybutyrate (2HB), which could be new non-invasive biomarkers for catabolic pathways of fatty acids/ketogenic amino acids, valine, leucine, and methionine/threonine/α-ketobutyrate, respectively. The four hydroxybutyrates (3HB, 3HIB, 3HMB, and 2HB) were extracted from 5 µl of saliva, converted to 2-pyridylmethyl (2PM) ester derivatives, and measured by liquid chromatography–tandem mass spectrometry in positive electrospray ionization mode. [^13^C_4_]3HB was used as an internal standard. The detection limits for the 2PM esters were <1 pg (7.9–9.6 fmol) on-column (signal-to-noise ratio = 3). Reproducibilities and recoveries of the hydroxybutyrates were validated according to one-way layout and polynomial equation, respectively. The variances between sample preparations and between measurements were calculated to be 0.45–5.28 and 0.54–3.45 %, respectively. Experiments performed using 5 µl of saliva spiked with 3.8–154.4 pmol of the four hydroxybutyrates gave recoveries of 98.5 to 108.8 %, with a mean recovery of 104.1 %. In vitro experiments in hepatocytes or skeletal muscle cells showed that addition of palmitic acid, valine, leucine or α-ketobutyrate to culture medium markedly increased the targeted hydroxybutyrate concentrations. The salivary concentration of each targeted hydroxybutyrate was positively correlated with that in serum, and the salivary levels were elevated in patients with liver cirrhosis, which is characterized by upregulated catabolism of lipids and amino acids. The proposed method is useful for quantification of salivary 3HB, 3HIB, 3HMB, and 2HB for monitoring of catabolic activities of amino acids and fatty acids.

**Electronic supplementary material:**

The online version of this article (doi:10.1186/s40064-015-1304-0) contains supplementary material, which is available to authorized users.

## Background

**Fig. 1:**
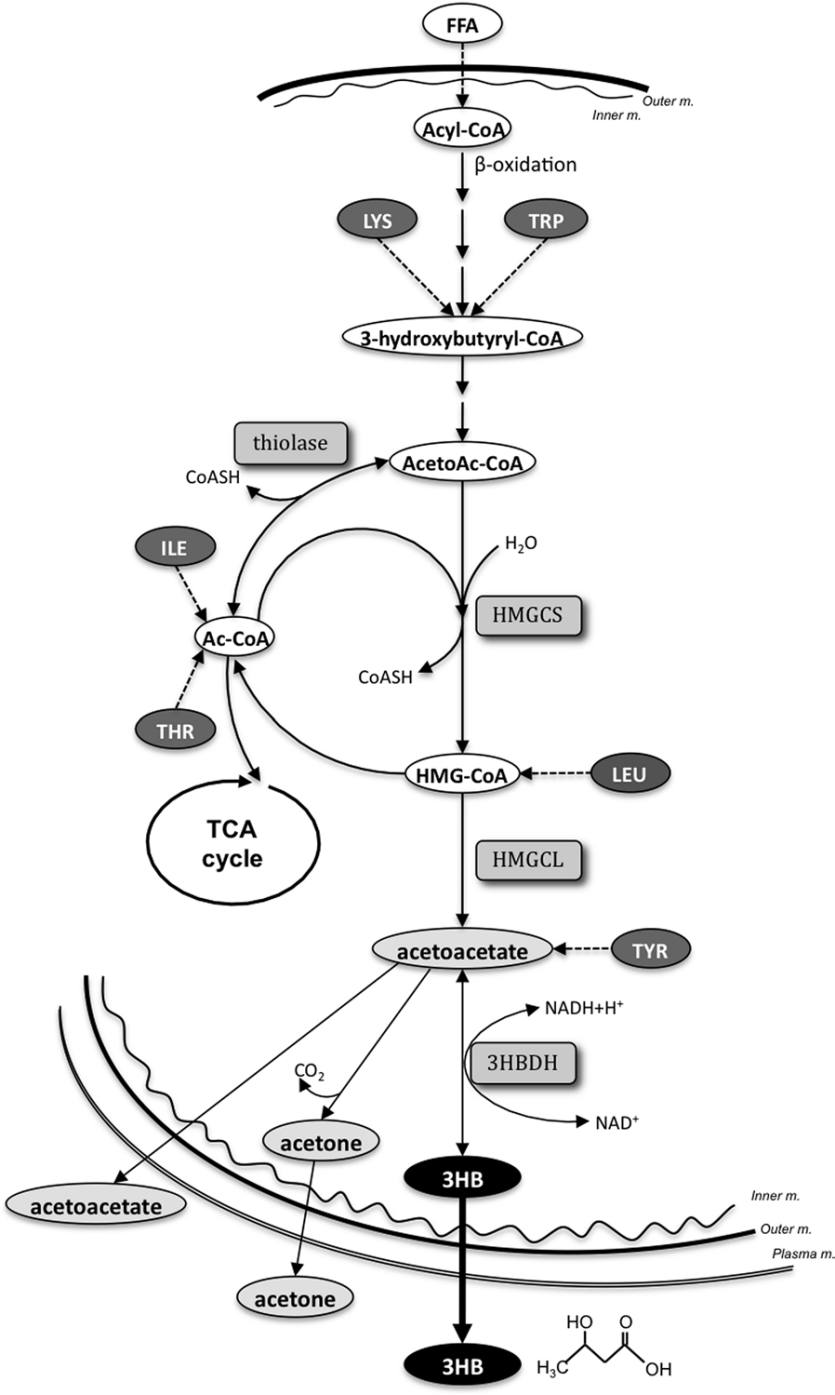
Productive pathways of 3HB from fatty acids and ketogenic amino acids. Acyl-CoA derived from fatty acids is metabolized to Ac-CoA through β-oxidation pathway in the mitochondria. Ac-CoA in the liver is further metabolized to ketone bodies (acetoacetate, 3HB, and acetone) to supply energy to non-hepatic tissues when blood glucose level is low. Ketone bodies can be also metabolized from ketogenic amino acids, which are metabolized to intermediates in fatty acids β-oxidation, Ac-CoA or HMG-CoA via the respective catabolic pathway. 3HB is the most stable in blood among the ketone bodies. *3HB* 3-hydroxybutyrate, *3HBDH* 3HB dehydrogenase, *Ac*-*CoA* acetyl-CoA, *AcetoAc*-*CoA* acetoacetyl-CoA, *FFA* fatty acids, *HMG-CoA* 3-hydroxy-3-methylglutaryl-CoA, *HMGCL* HMG-CoA lyase, *HMGCS* HMG-CoA synthase, *ILE* isoleucine, *Inner m.* mitochondria inner membrane, *LEU* leucine, *LYS* lysine, *Outer m.* mitochondrial outer membrane, *Plasma m.* plasma membrane, *THR* threonine, *TRP* tryptophan, *TYR* tyrosine

Energy sources fluctuate among carbohydrates, lipids, and amino acids depending on nutritional and metabolic status, such as feeding, fasting, and exercise. Under conditions of fasting, endurance exercise, malnutrition or metabolic disorders including diabetes mellitus (Gaster 2009; Adams et al. 2009) and chronic liver diseases (Syed et al. 2010), catabolism of fatty acids and amino acids in the liver and skeletal muscles is stimulated to compensate for the lack of glucose supply. Fatty acids are oxidized to acetyl-CoA in the mitochondrion (β-oxidation) and used in the TCA cycle, and excess acetyl-CoA in the liver is further metabolized to ketone bodies to supply energy to non-hepatic tissues, mainly brain and skeletal muscles (Fig. 1) (Laffel 1999). Amino acids are also an important energy source under conditions lacking glucose, and ketone bodies could be produced from ketogenic amino acids in the liver in such metabolic status (Fig. 1). Because 3-hydroxybutyrate (3HB) is the most stable ketone body in blood, serum 3HB concentration reflects fatty acid β-oxidation as well as ketogenic amino acids catabolism in the liver (Robinson and Williamson 1980).

**Fig. 2:**
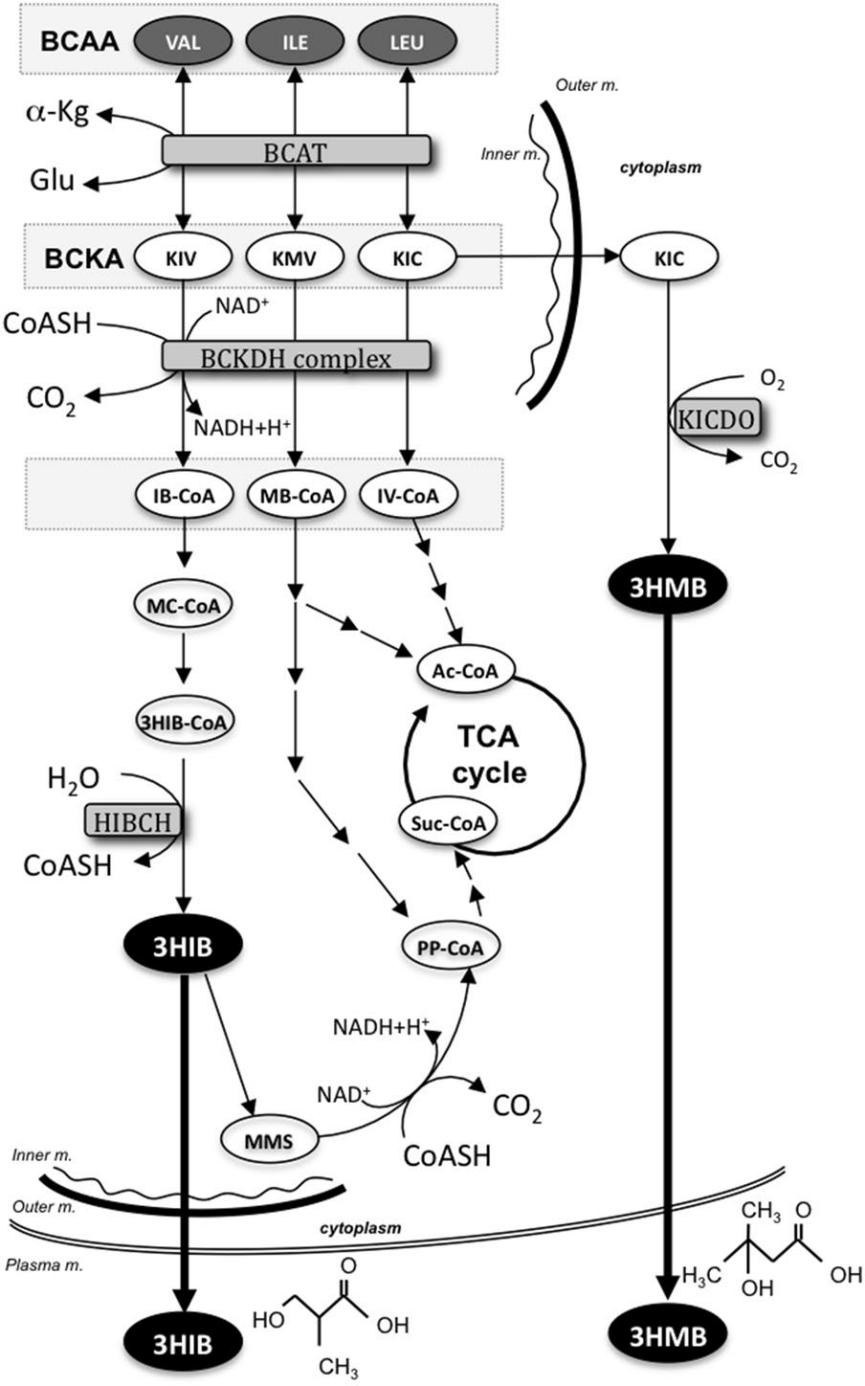
Productive pathways of 3HIB from VAL and 3HMB from LEU in BCAA metabolism. BCAAs are metabolized in the mitochondrion and the first two steps are catalyzed by common enzymes; first, BCAAs are deaminated to the respective BCKA by BCAT, and then the BCKAs are converted to the respective CoA-derivatives by the BCKDH complex, which is the rate-limiting enzyme in BCAA catabolism. The respective CoA derivatives are finally metabolized to Ac-CoA and/or Suc-CoA. 3HIB is an intermediate of VAL catabolism that is released from 3HIB-CoA by HIBCH in the mitochondrion. The small molecule 3HIB leaks into extracellular fluid. 3HMB is synthesized from KIC by KICDO in the cytoplasm as a BCKA derived from LEU. 3HMB is also detectable in extracellular fluid. *3HIB* 3-hydroxyisobutyrate, *3HIB*-*CoA* 3-hydroxyisobutyryl-CoA, *α*-*Kg* α-ketoglutaric acid, *Ac*-*CoA* acetyl-CoA, *BCAA* branched-chain amino acid, *BCAT* branched-chain aminotransferase, *BCKA* branched-chain α-keto acid, *BCKDH complex* BCKA dehydrogenase complex, *GLU* glutamate, *KIC* α-ketoisocaproic acid, *KICDO* KIC dioxygenase, *KMV* α-keto-β-methylvaleric acid, *KIV* α-ketoisovaleric acid, *HIBCH* 3HIB-CoA hydrolase, *IB*-*CoA* isobutyryl-CoA, *ILE* isoleucine, *Inner m.* mitochondria inner membrane, *IV*-*CoA* isovaleryl-CoA, *LEU* leucine, *MB*-*CoA* α-methylbutyryl-CoA, *MC*-*CoA* methacrylyl-CoA, *MMS* methylmalonate semialdehyde, *Outer m.* mitochondrial outer membrane, *Plasma m.* plasma membrane, *PP*-*CoA* propionyl-CoA, *Suc*-*CoA* succinyl-CoA, *VAL* valine

In humans, branched-chain amino acids (BCAAs), valine (VAL), leucine (LEU) and isoleucine (ILE), are metabolized to succinyl-CoA or acetyl-CoA exclusively in skeletal muscle mitochondria and used in the TCA cycle (Shimomura et al. 2006; Platell et al. 2000; Rennie et al. 2006; Kong et al. 2012). The first two steps of the BCAA metabolic pathways are conversion of BCAAs to their CoA-derivatives, catalyzed by common mitochondrial enzymes. The CoA-derivatives of BCAAs are finally metabolized to succinyl-CoA or acetyl-CoA by their own pathways, but CoA is released from 3-hydroxyisobutyryl-CoA only in the VAL catabolic pathway (Fig. 2). CoA-free 3-hydroxyisobutyrate (3HIB) is then released from mitochondria into extracellular fluid (Letto et al. 1986). Therefore, 3HIB is a possible biomarker of VAL catabolism in skeletal muscle (Avogaro and Bier 1989). LEU is transaminated to α-ketoisocaproate (KIC) in the first step of the mitochondrial catabolic pathway, and approximately 5 % of KIC is transferred to cytoplasm and metabolized to 3-hydroxy-3-methylbutyrate (3HMB) by KIC dioxygenase (Fig. 2) (Sabourin and Bieber 1983). The synthesis of 3HMB is not directly associated with energy production (Zanchi et al. 2011), but this molecule can also pass through the plasma membrane and may be a biomarker for LEU catabolism in skeletal muscle.

**Fig. 3:**
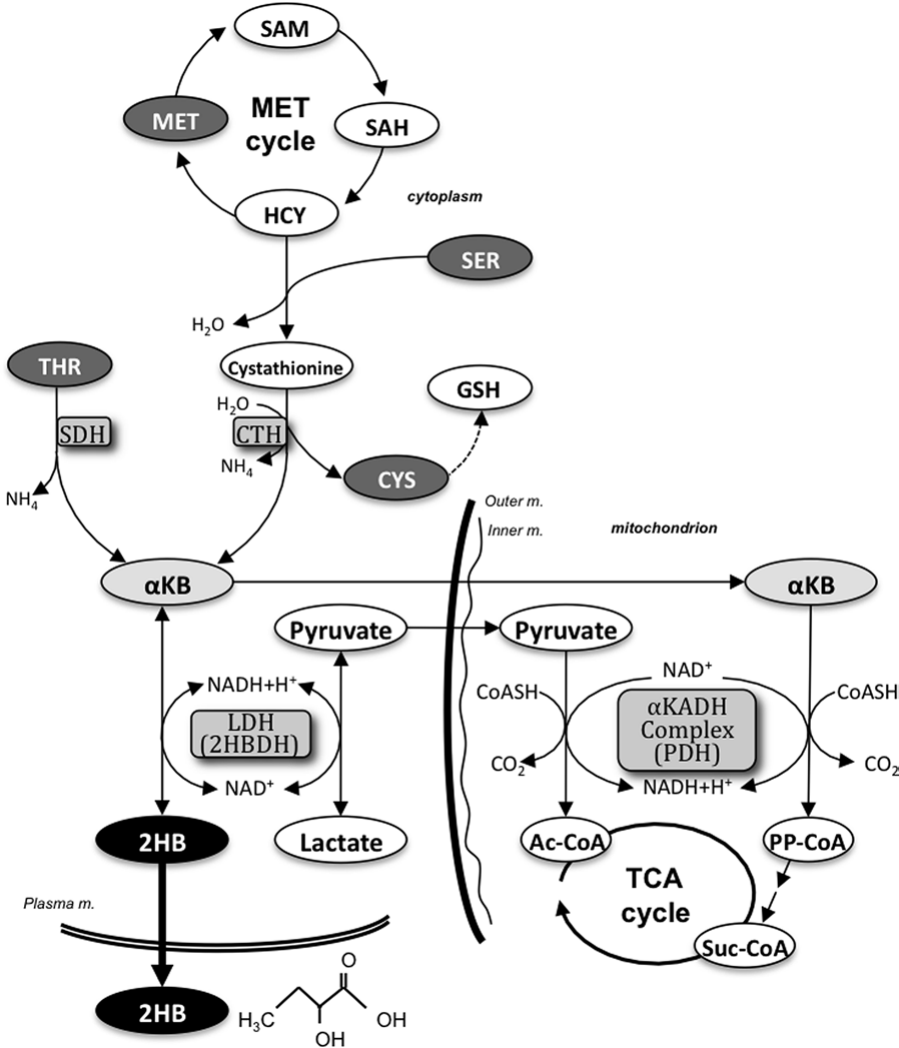
Productive pathway of 2HB from αKB in the sulfur-containing amino acid metabolic pathway. Cystathionine, a metabolite of homocysteine, is metabolized to αKB and CYS. αKB is transported into the mitochondrion and converted to PP-CoA, a precursor of Suc-CoA, and is also reversibly converted to 2HB by 2HBDH. 2HB leaks into extracellular fluid. *2HB* 2-hydroxybutyrate, *2HBDH* 2HB dehydrogenase, α*KB* α-ketobutyrate, α*KADH* α-keto acid dehydrogenase, *CYS* cysteine, *GSH* glutathione, *HCY* homocysteine, *Inner* *m.* mitochondrial inner membrane, *LDH* lactate dehydrogenase, *MET* methionine, *Outer m.* mitochondrial outer membrane, *PDH* pyruvate dehydrogenase, *Plasma m.* plasma membrane, *PP*-*CoA* propionyl-CoA, *SAM* *S*-adenosyl methionine, *SAH* *S*-adenosyl homocysteine, *SER* serine, *SDH* serine/threonine dehydrase, *Suc*-*CoA* succinyl-CoA, *THR* threonine

In addition to production in the VAL and LEU catabolic pathways, succinyl-CoA is produced from α-ketobutyrate (αKB), which is generated when cysteine is synthesized from cystathionine by γ-cystathionase (CTH) in the methionine (MET) pathway or from threonine (THR) by serine/threonine dehydratase (SDH) (Fig. 3) (Stipanuk 2004; Greenberg et al. 1967; Yang and Roth 1985). In this pathway, some cytoplasmic αKB is further reduced to 2-hydroxybutyrate (2HB) and this compound is released into extracellular fluid. It is uncertain if increased serum 2HB directly reflects upregulated catabolism of MET/THR, but metabolome analyses have shown that 2HB is a significant biomarker associated with early stage insulin resistance (Syed Ikmal et al. 2013; Gall et al. 2010; Ferrannini et al. 2013; Tripathy et al. 2015).

Non-invasive evaluation of metabolic status is increasingly important. Saliva has been used as a non-invasive sample in clinical examination, therapy and sport because it contains various metabolites and can be self-collected easily and safely. Plasma 3HB, 3HIB and 3HMB are measured by gas chromatography–mass spectrometry (GC–MS) (Des Rosiers et al. 1988; Avogaro and Bier 1989; Nissen et al. 1990) and plasma 2HB, 3HB, and 3HMB by liquid chromatography–tandem mass spectrometry (LC–MS/MS) in negative electrospray ionization (N-ESI) mode (Deshpande et al. 2013; Sorensen et al. 2013; Gall et al. 2010; Ferrannini et al. 2013). However, these methods are not sufficiently sensitive for measurement of 2HB, 3HB, and 3HMB in saliva. We have previously converted another organic acid, malonic acid, into a di-(1-methyl-3-piperidinyl)malonic acid derivative for measurement by LC–MS/MS in positive electrospray ionization (P-ESI) mode (Honda et al. 2009). The introduction of a tertiary amine moiety in malonate promoted protonation and markedly increased the sensitivity. Derivatization has also been used to quantify salivary 3HB (Tsutsui et al. 2012). In the current study, we converted salivary 3HB, 3HIB, 3HMB, and 2HB into 2-pyridylmethyl (2PM) ester derivatives for simultaneous quantification using LC-P-ESI–MS/MS. In vivo and in vitro experiments showed that the levels of salivary 3HB, 3HIB, 3HMB, and 2HB reflect their serum concentrations, which indicates that these molecules could be useful biomarkers for monitoring of catabolic pathways of amino acids and fatty acids.

## Methods

### Chemicals

Sodium 3HIB, sodium D-3HB, 3HMB, αKB, fenofibrate and fatty acid-free BSA were purchased from Sigma-Aldrich (St. Louis, MO, USA). Sodium DL-3HB-^13^C_4_ was obtained from Taiyo Nippon Sanso Co. (Tokyo, Japan) and sodium DL-2HB, 2-pyridinemethanol and 2-methyl-6-nitrobenzoic anhydride were from Tokyo Kasei Kogyo (Tokyo, Japan). VAL, LEU, ILE, L-carnitine, palmitic acid and 4-dimethylaminopyridine were purchased from Wako Pure Chemical Industries (Osaka, Japan). Amino acid-free medium (Zero medium) was kindly supplied by Ajinomoto Pharmaceuticals Co., Ltd. (Tokyo, Japan). All other reagents for cell culture experiments were purchased from Thermo Fisher Scientific (Gibco^®^, Waltham, MA, USA). Solvents used for analysis were of analytical grade.

### Sample collection

Saliva was collected from healthy volunteers (*n* = 15) and patients with liver cirrhosis (*n* = 22) by passive drool into a collection tube after mouth was washed out with water several times, and was immediately frozen at −20 °C until analysis. Blood and saliva were simultaneously collected from three healthy volunteers several times on different days to compare the concentrations of 3HB, 3HIB, 3HMB, and 2HB in saliva and serum. Blood samples were coagulated and centrifuged at 1500×*g* for 10 min, and serum was stored at −20 °C until analysis. In addition, to examine the influence of standing sample at room temperature (RT) on stability of four examined hydroxybutyrates, collected saliva samples from healthy volunteers (*n* = 4) were aliquoted into a microcentrifuge tube (1.5 mL, Eppendorf, Hamburg, Germany) and kept in incubator set at 25 °C for 0, 1, 2, 4, 6, 10, and 24 h, and then, transferred at −20 °C.

Informed consent was obtained from all subjects and the study protocol was approved by the Ethics Committee of Tokyo Medical University Ibaraki Medical Center (#12–34).

### Sample preparation

Before analysis, saliva was thawed at RT and centrifuged at 3000×*g* for 15 min at 4 °C to remove denatured mucins and food particles (Tsutsui et al. 2012). Five µL of the supernatant of saliva, serum, or culture medium was placed in the microcentrifuge tube, and 769 pmol (100 ng) of sodium DL-3HB-^13^C_4_ in 100 μL of acetonitrile–water (19:1, *v/v*) was added as an internal standard. The sample tube was vortexed for 1 min and centrifuged at 2000×*g* for 1 min. After centrifugation, deproteinized clear liquid phase was collected and evaporated to dryness at 55 °C under a nitrogen stream. Conversion of 3HB, 3HIB, 3HMB, and 2HB into 2PM ester derivatives was performed with some modifications of Shiina’s method for synthesis of carboxylic esters (Shiina et al. 2002). The reagent mixture consisted of 2-methyl-6-nitrobenzoic anhydride (67 mg), 4-dimethylaminopyridine (20 mg), pyridine (900 μL) and 2-pyridinemethanol (100 μL). The freshly prepared reagent mixture (50 μL) was added to the sample extracts and the resulting mixture was allowed to stand at RT for 30 min. After addition of 1 mL of diethyl ether, the mixture was vortexed for 1 min and centrifuged at 700×*g* for 1 min. The clear supernatant was collected and evaporated at 55 °C under nitrogen. The residue was redissolved in 100 μL of 1 % formic acid in water and centrifuged again at 700×*g* for 1 min. The supernatant was collected and a 2-µL aliquot was injected into the LC–MS/MS system.

### Determination of 2PM-3HB/3HIB/3HMB/2HB by LC-P-ESI–MS/MS

The LC–ESI–MS/MS system consisted of a TSQ Vantage triple stage quadrupole mass spectrometer (Thermo Fisher Scientific, Waltham, MA, USA) equipped with an HESI-II probe and a Prominence ultra-fast liquid chromatography (UFLC) system (Shimadzu, Kyoto, Japan). Chromatographic separation was performed using a Hypersil GOLD aQ column (150 × 2.1 mm, 3 μm, Thermo Fisher Scientific) at 40 °C. Initially, the mobile phase was acetonitrile–water (1:19, *v/v*) containing 0.2 % formic acid and was used at a flow rate of 300 µL/min for 5 min. After 5 min, the mobile phase was switched to 0.2 % formic acid in acetonitrile at a flow rate of 300 μL/min for an additional 7 min. The general MS/MS conditions were as follows: spray voltage, 3000 V; vaporizer temperature, 450 °C; sheath gas (nitrogen) pressure, 50 psi; auxiliary gas (nitrogen) flow, 15 arbitrary units; ion transfer capillary temperature, 220 °C; collision gas (argon) pressure, 1.0 mTorr; collision energy, 15 V; and ion polarity, positive; and selected reaction monitoring (SRM), *m/z* 196 → *m/z* 110 for 2PM-3HB, 2PM-3HIB and 2PM-2HB, *m/z* 210 → *m/z* 192 for 2PM-3HMB, and *m/z* 200 → *m/z* 110 for 2PM-[^13^C_4_]3HB.

### Calibration curves

Stock solutions of sodium D-3HB (40 ng/20 μL), sodium 3HIB (40 ng/20 μL), 3HMB (40 ng/20 μL) and sodium DL-2HB (40 ng/20 μL) for construction of calibration curves were prepared in acetonitrile–water (19:1, *v/v*). These solutions were further diluted with the same solvent to give a series of working standard solutions (1–200 ng/100 μL for 3HB, 0.1–40 ng/100 μL for 3HIB, 0.1–20 ng/100 μL for 3HMB and 2HB). Sodium DL-3HΒ-^13^C_4_ (100 ng) was added as an internal standard to each standard solution and the mixture was evaporated to dryness, derivatized and quantified, as described above.

### Cell culture

Differentiated, nontransformed AML12 cells from transforming growth factor α-overexpressing transgenic mice (Wu et al. 1994) (ATCC, Manassas, VA, USA) were used as a non-tumor mouse hepatocyte cell line. AML12 cells were cultured on a 24-well plate until confluent (Honda et al. 2011a). For evaluation of hepatic β-oxidation, the growth medium was replaced by DMEM containing 1.0 g/L glucose, 1 mM l-carnitine and 200 µM palmitic acid dissolved in 10 % (*w/v*) fatty acid-free BSA with or without 100 µM fenofibrate, a synthetic peroxisome proliferator-activated receptor α (PPARα) ligand. After 24 h, the 3HB level in the culture medium was measured. Confluent cells seeded on a 24-well plate were also exposed to 20 mM MET, cystathionine, THR or αKB in Zero medium containing 925 mg/L NaHCO_3_, 4.5 g/L glucose and 1 mM sodium pyruvate (Honda et al. 2011b) for 24 h. Serine (20 mM) was also added to the medium containing MET. After exposure, the 2HB level in the culture medium was measured.

Growth of human primary skeletal muscle myoblasts isolated from the rectus abdominus muscle (ZenBio, Inc., NC, USA) and differentiation into myotubes were carried out on a 6-well plate following the recommended procedure of the manufacturer (ZenBio, Inc.). The differentiation medium was replaced by Zero medium containing 2 mM VAL, LEU or ILE (Honda et al. 2011b). 3HIB and 3HMB levels in the medium were measured after 24 h. All cells were incubated at 37 °C in a humidified incubator containing 5 % CO_2_ and 95 % air.

### Statistics

The linearity of calibration curves was analyzed by simple linear regression. Reproducibility was analyzed by one-way ANOVA. The estimated amount ± 95 % confidence limit was obtained as an index of precision (Taguchi 1986). To calculate these values, orthogonal regression analysis was performed in a recovery study. The significance of differences between the results in different groups was evaluated by unpaired Student two-tailed *t* test or non-parametric Mann–Whitney *U* test. Data are reported as mean ± SEM. For all analyses, significance was accepted at *P* < 0.05. All statistical analyses were carried out using JMP software (SAS Institute Inc., Cary, NC, USA).

## Results

### MS, MS/MS and SRM

**Fig. 4:**
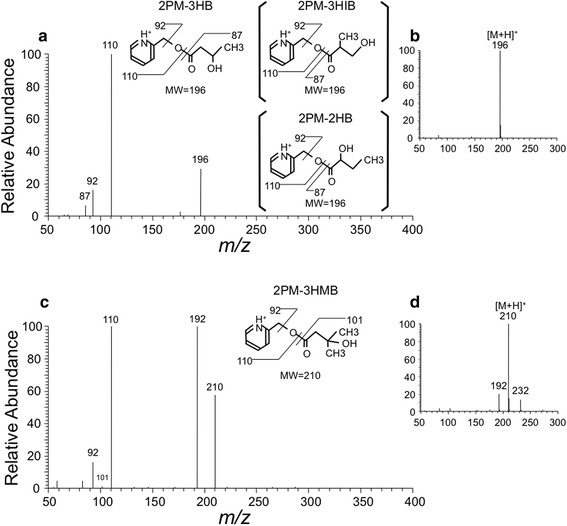
Typical P-ESI mass spectra (**b**, **d**) and product ion mass spectra (**a**, **c**) for 2PM-3HB (*m/z* 196) and 2PM-3HMB (*m/z* 210). The mass spectra of 2PM-3HIB and 2PM-2HB were almost the same as 2PM-3HB. The LC–MS/MS conditions are described in the “Methods”. *P*-*ESI* positive electrospray ionization, *2PM* 2-pyridylmethyl. See legends of Figs. 1, 2, and 3 for other abbreviations

Typical P-ESI mass spectra for the 2PM esters of 3HB and 3HMB are shown in Fig. 4b, d. 2PM-3HB and 2PM-3HMB exhibited [M + H]^+^ ions at *m/z* 196 and *m/z* 210, respectively, as the base peaks. In MS/MS spectra using these base peaks as precursor ions, the [C_5_H_4_NCH_2_OH + H]^+^ ion was observed at *m/z* 110 as the most prominent peak (Fig. 4a, c). The [M-H_2_O + H]^+^ ion at *m/z* 192 was the second most prominent peak for 2PM-3HMB (Fig. 4c). The mass spectra and MS/MS spectra for 2PM-3HIB and 2PM-2HB were similar to those for 2PM-3HB. SRM chromatograms obtained for *m/z* 210 → *m/z* 110 and *m/z* 210 → *m/z* 192 were compared for determination of 2PM-3HMB, and the latter showed a better signal-to-noise ratio (S/N) for biological samples. The detection limits of the 2PM esters of the four hydroxybutyrates by SRM were <1 pg (7.9–9.6 fmol) on-column (S/N = 3).

### Calibration curves

Calibration curves were established for 3HB, 3HIB, 3HMB and 2HB (Additional file 1: Fig. S1). Different amounts of authentic hydroxybutyrates (0–200 ng for 3HB, 0–40 ng for 3HIB, 0–20 ng for 3HMB and 2HB) were mixed with 100 ng (769 pmol) of [^13^C_4_]3HB as internal standard. The amount of each hydroxybutyrate and the peak-area ratio to the internal standard measured by SRM were plotted on the abscissa and ordinate, respectively. Good linearity was found based on simple linear regression: 3HB: y = 0.0092x + 0.0020, *n* = 8, r^2^ = 0.9998, *P* < 0.0001; 3HIB: y = 0.0211x + 0.0032, *n* = 7, r^2^ = 0.9998, *P* < 0.0001; 3HMB: y = 0.0298x + 0.0043, *n* = 6, r^2^ = 0.9991, *P* < 0.0001; 2HB: y = 0.0322x + 0.0038, *n* = 6, r^2^ = 0.9993, *P* < 0.0001.

### Representative SRM chromatograms

**Fig. 5:**
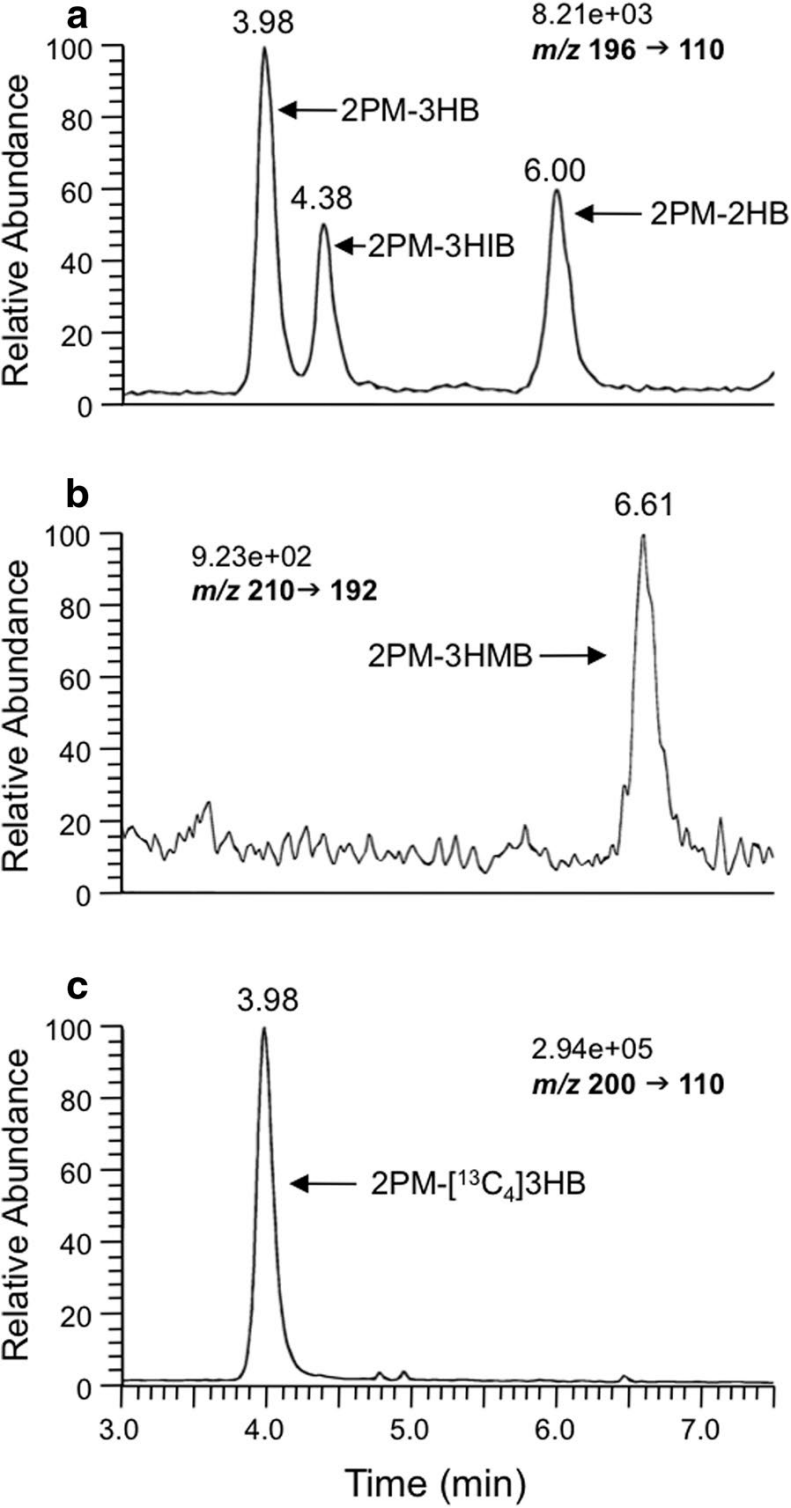
Representative SRM chromatograms of 2PM-derivatized 3HB, 2HIB, 3HMB, and 2HB in 5 µL of saliva from a healthy human. **a** *m/z* 196 g *m/z* 110 for 2PM-3HB, 2PM-3HIB and 2PM-2HB. **b** *m/z* 210 g *m/z* 192 for 2PM-3HMB. **c** *m/z* 200 g *m/z* 110 for 2PM-[^13^C_4_]3HB. The peaks for 3HB, 3HIB, 2HB and 3HMB in chromatograms correspond to 650 fmol (6.5 µM), 350 fmol (3.5 µM), 450 fmol (4.5 µM) and 70 fmol (0.7 µM), respectively. The numbers above the SRM ion pair represent the full scale of the chromatogram. *SRM* selected reaction monitoring; and see legends of Figs. 1, 2, 3, 4 for other abbreviations

Selected reaction monitoring chromatograms of 2PM esters of 3HB, 3HIB, 3HMB, 2HB and [^13^C_4_]3HB obtained with 5 μL of saliva from a healthy human subject are shown in Fig. 5. The peak-area ratio of each 2PM ester to 2PM-[^13^C_4_]3HB was calculated from the chromatograms and the amount was determined using this ratio on the calibration curve. The peaks for 2PM-3HB, 2PM-3HIB, 2PM-2HB and 2PM-3HMB on the chromatogram correspond to ~650 fmol (6.5 µM), ~350 fmol (3.5 µM), ~450 fmol (4.5 µM) and ~70 fmol (0.7 µM), respectively.

### Precision and accuracy of the method

The following studies were performed to determine the precision and accuracy of the method using saliva from a healthy human subject. Reproducibility was investigated by analysis of quadruplicate samples by LC-P-ESI-SRM in triplicate (Table 1, Additional file 2: Tables S1, S2). The results were analyzed by one-way ANOVA, in which analytical errors were divided into two sources: sample preparation and SRM measurement. Variances were not considered to be attributable to the sample preparation because the errors during sample preparation were not significantly larger than those between the measurements. The inter-assay coefficients of variation for between- and within-sample variations of the four hydroxybutyrates were 0.45–5.28 and 0.54–3.45 %, respectively.

**Table 1:** Reproducibility of the quantification of each hydroxybutyrate in human saliva

Hydroxybutyrate	Mean ± SD (n = 12) (pmol)	Relative SD	
		Sample preparation (%)	Error (SRM) (%)
3HB	56.0 ± 0.30	0.48	0.54
3HIB	49.0 ± 0.36	0.45	0.79
3HMB	3.7 ± 0.10	1.70	2.98
2HB	19.5 ± 0.84	5.28	3.45

Each butyrate was quantified in 5 µL of saliva collected from healthy subjects. Four samples were prepared and quantified in triplicate by HPLC–MS/MS. The results were analyzed by a one-way layout, in which the analytical errors were divided into two sources: sample preparation and SRM measurement

*3HB* 3-hydroxybutyrate, *3HIB* 3-hydroxyisobutyrate, *3HMB* 3-hydroxy-3-methylbutyrate, *2HB* 2-hydroxybutyrate

In recovery experiments, known amounts of each targeted hydroxybutyrate (a, 2a, 3a; a = 51.2 pmol for 3HB, 50.0 pmol for 3HIB, 3.8 pmol for 3HMB, and 19.2 pmol for 2HB) were spiked into 5-μL aliquots of saliva samples (*n* = 2). After clean-up and derivatization, SRM was conducted in triplicate for each sample. The mean recoveries were 103.7 % for 3HB (102.1–104.6 %), 99.6 % for 3HIB (98.5–100.7 %), 106.3 % for 3HMB (104.8–107.9 %), and 106.8 % for 2HB (108.7–118.8 %) (Table 2). The amount of each endogenous hydroxybutyrate found in unspiked 5-μL saliva aliquots was within the 95 % confidence limit for the respective estimated amount calculated by orthogonal regression analysis, which also constituted an index for the precision and accuracy of the method.

**Table 2:** Recovery of each hydroxybutyrate from human saliva

Hydroxybutyrate	Amount added (pmol)	Average recovery^a^ [mean ± SD (n)] (%)
3HB	51.2	103.4 ± 3.6 (6)
	103.2	104.6 ± 1.6 (6)
	154.4	102.1 ± 1.1 (6)
3HIB	50.0	98.5 ± 2.0 (6)
	100.0	99.7 ± 1.0 (6)
	150.0	100.7 ± 1.1 (6)
3HMB	3.8	104.8 ± 1.5 (6)
	7.6	107.7 ± 0.6 (6)
	11.4	107.9 ± 0.9 (6)
2HB	19.2	108.8 ± 5.4 (6)
	38.4	118.8 ± 2.2 (6)
	57.6	108.7 ± 2.5 (6)

Known amounts of each hydroxybutyrate were spiked into 5 µL of human saliva collected from healthy subjects before sample preparation

^a^Recovery (%) = (amount found − $\bar{X}_{0}$)/amount added × 100; $\bar{X}_{0}$ value was obtained from Table 1

### Cell culture experiments

**Fig. 6:**
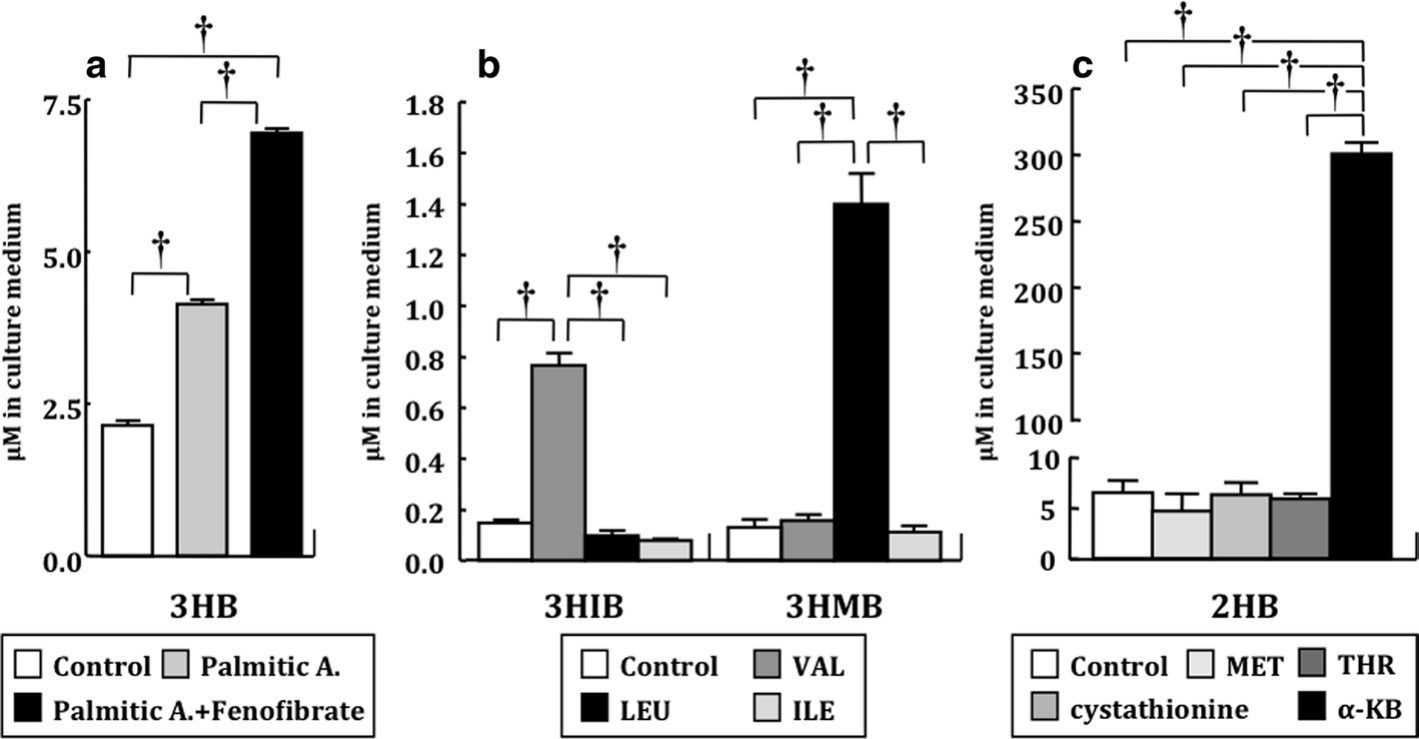
Concentrations of 3HB, 3HIB, 3HMB, and 2HB in cell culture media after addition of precursor compounds. **a** AML12 cells were treated with 200 µM palmitic acid or 200 µM palmitic acid plus 100 µM fenofibrate for 24 h. 3HB concentrations were compared with that in untreated cells. **b** Myotubes were treated with 2 mM VAL, LEU or ILE for 24 h. 3HIB and 3HMB concentrations were compared with those in untreated cells. **c** AML12 cells were treated with 2 mM MET plus SER, cystathionine, THR or αKB for 24 h, and 2HB concentrations were compared among these conditions. *Palmitic A* palmitic acid. See legends of Figs. 1, 2, and 3 for other abbreviations. Data are from four independent experiments and are shown as mean ± SEM. Statistical analysis by one-way ANOVA multiple comparison and a post hoc Bonferroni multiple comparison test. ^†^ *P* < 0.001. All ANOVA *P* values were <0.0001

3HB, 3HIB, 3HMB, and 2HB levels in culture medium were measured following treatment of cells with different compounds. The level of 3HB, a possible marker of hepatic fatty acid β-oxidation, was significantly increased when hepatocytes were exposed to palmitic acid (Fig. 6a). Addition of fenofibrate with palmitic acid stimulated accumulation of 3HB in the medium. Treatment of myotubes with VAL and LEU caused significant elevation of the concentrations of 3HIB and 3HMB, respectively, in the medium compared with untreated myotubes or treatment with other BCAAs (Fig. 6b). Addition of other amino acids to the medium did not cause a significant change in the 3HIB or 3HMB level in the culture medium (data not shown). In exposure of hepatocytes to αKB or its precursors (MET, THR or cystathionine), only αKB led to accumulation of 2HB in the medium (Fig. 6c).

### Human studies

**Fig. 7:**
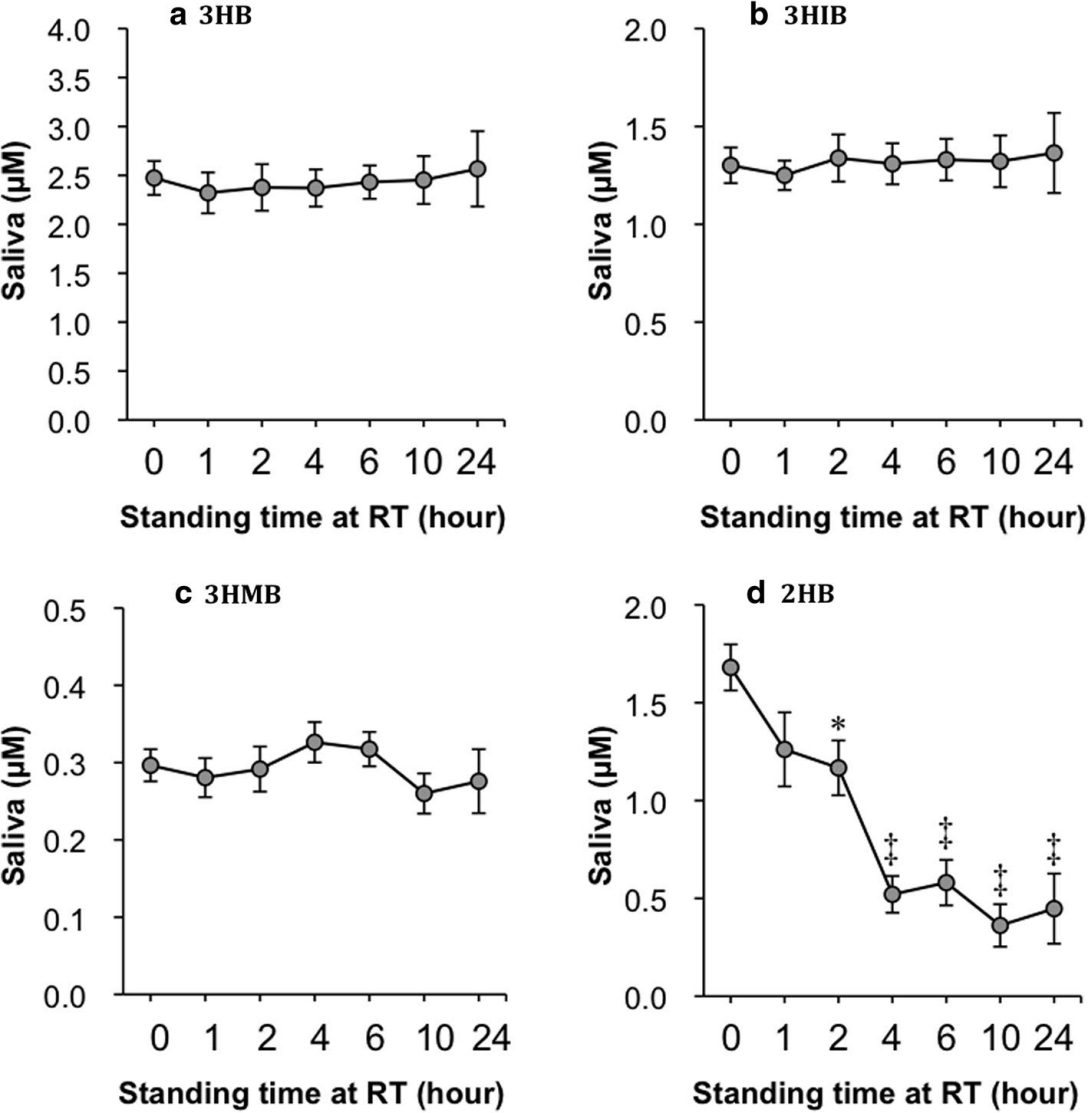
Influence of standing saliva samples at RT on stability of 3HB (**a**), 3HIB (**b**), 3HMB (**c**), and 2HB (**d**). Saliva samples collected from healthy volunteers (*n* = 4) were aliquoted into seven microcentrifuge tubes and standing at RT for various times (0, 1, 2, 4, 6, 10, and 24 h). Thereafter, the saliva samples were stored at −20 °C until analysis. *RT* room temperature. See legends of Figs. 1, 2, and 3 for other abbreviations. Data are shown as mean ± SEM. Statistical analysis by one-way ANOVA multiple comparison and a post hoc Dunnett’s multiple comparison test. ANOVA *P* values are **a** *P* = 0.9949, **b** *P* = 0.9968, **c** *P* = 0.7058, and **d** *P* < 0.0001. *P < 0.05, ^‡^ *P* < 0.0001 vs. 0-time control

Saliva collected from healthy volunteers was stood at RT (25 °C) for various times, and then, stored at −20 °C until analysis. After standing at RT, 3HB and 3HIB concentrations in saliva were unchanged for up to 24 h (Fig. 7). Similarly, 3HMB concentration in saliva was mostly kept for up to 24 h, although some variations were observed. On the other hand, 2HB concentration in saliva begun to decrease from an hour after standing at RT, and decreased significantly over one-third fold after four hours (Fig. 7). Therefore, saliva samples were stored at −20 °C immediately after collection in all other experiments.

**Fig. 8:**
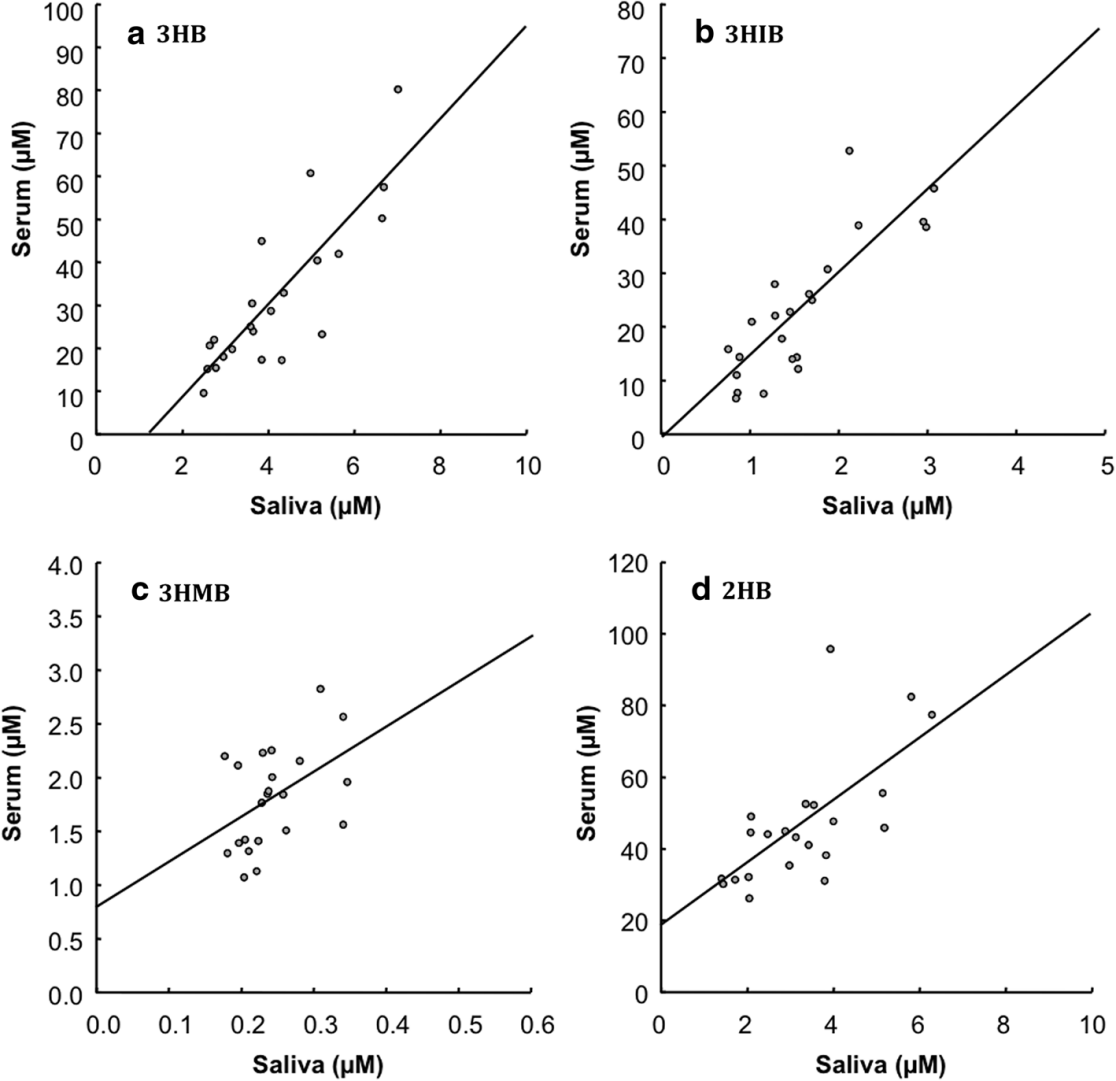
Correlation of 3HB, 3HIB, 3HMB, and 2HB concentrations between serum and saliva. **a** *3HB* 3-hydroxybutyrate, y = 10.8x − 13.6 (r^2^ = 0.7034, *P* < 0.0001), **b** *3HIB* 3-hydroxyisobutyrate, y = 15.4x − 1.2 (r^2^ = 0.6930, *P* < 0.0001), **c** *3HMB* 3-hydroxy-3-methylbutyrate, y = 4.2x − 0.8 (r^2^ = 0.2088, *P* < 0.05), **d** *2HB* 2-hydroxybutyrate, y = 8.7x − 18.2 (r^2^ = 0.4599, *P* < 0.001)

**Fig. 9:**
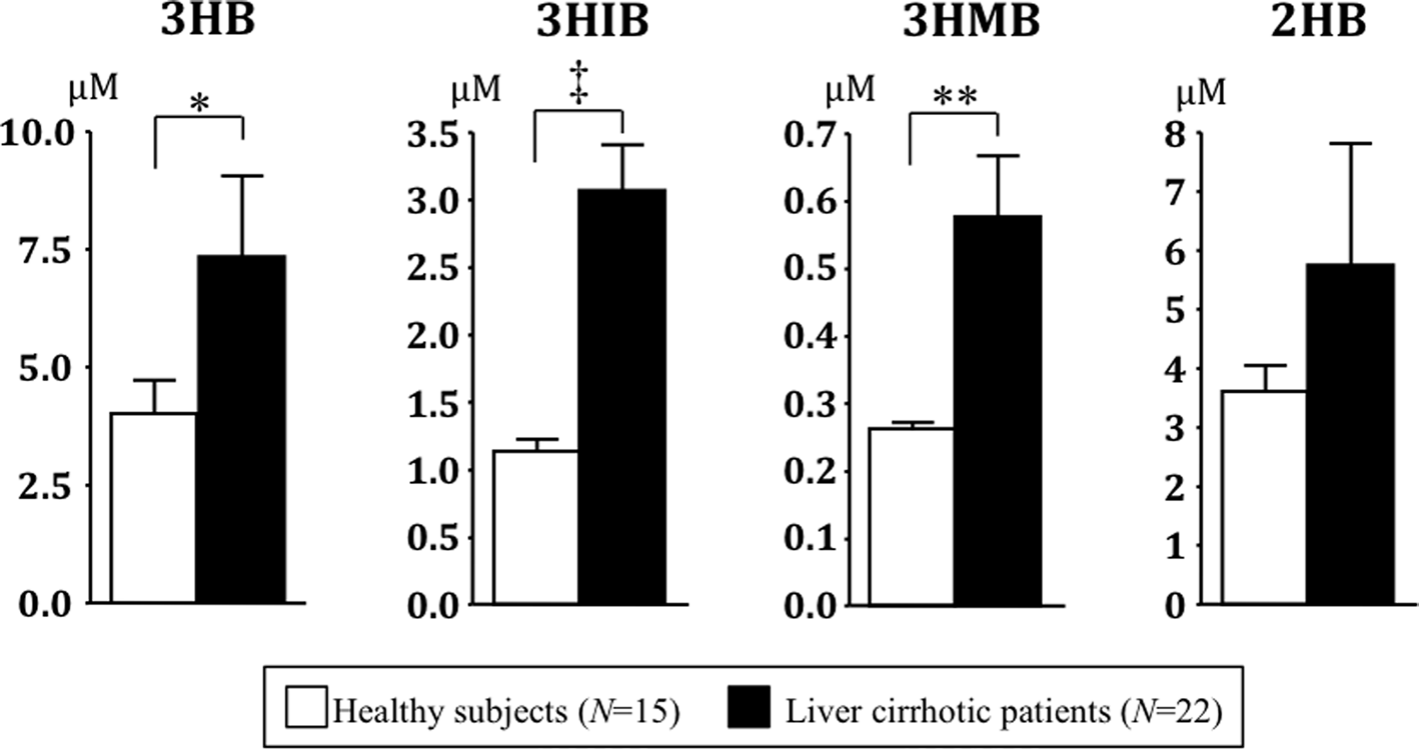
Concentrations of 3HB, 3HIB, 3HMB, and 2HB in saliva from patients with liver cirrhosis and healthy controls. Data are expressed as mean ± SEM. Statistical analysis by unpaired Student *t* test or non-parametric Mann–Whitney *U* test. * *P* < 0.05, ** *P* < 0.01, ^‡^ *P* < 0.0001. See legends of Figs. 1, 2, and 3 for abbreviations

Serum and saliva were collected from three healthy subjects repeatedly on different days. Significant correlations of the serum and saliva concentrations were found for all four hydroxybutyrates (3HB: r^2^ = 0.7034, *P* < 0.0001; 3HIB: r^2^ = 0.6930, *P* < 0.0001; 3HMB: r^2^ = 0.2088, *P* < 0.05; and 2HB: r^2^ = 0.4599, *P* < 0.001) (Fig. 8). These results suggest that the salivary levels reflect the serum levels of these examined hydroxybutyrates. The levels of 3HB, 3HIB, and 3HMB in saliva were significantly higher in patients with liver cirrhosis compared with healthy controls (Fig. 9); and that of 2HB tended to be higher in liver cirrhosis, but the difference was not significant.

## Discussion

We established a sensitive and specific new LC-P-ESI–MS/MS method for simultaneous quantification of four hydroxybutyrates (3HB, 3HIB, 3HMB, and 2HB) in human saliva, serum, and cell culture medium. Several methods for quantification of some hydroxybutyrates using LC-N-ESI–MS/MS have been developed (Deshpande et al. 2013; Sorensen et al. 2013). However, these methods have detection limits for 3HMB and 3HB of 0.3 µM (Deshpande et al. 2013) and 3 µM (Sorensen et al. 2013), respectively, and a >100-µL blood sample is required for quantification. The concentrations of the four examined hydroxybutyrates in saliva are 0.7–6.5 µM (Fig. 5) and quantification of these compounds in a small volume of saliva is difficult. Indeed, our preliminary study showed that only trace amounts of the four hydroxybutyrates in saliva were detected by LC-N-ESI–MS/MS without derivatization.

Conversion of the four hydroxybutyrates into 2PM ester derivatives gave excellent peak shapes, [M + H]^+^ ions as base peaks in P-ESI–MS, and detection limits by SRM that were approximately 50 times lower than those for underivatized hydroxybutyrates in N-ESI mode (data not shown). Using this method, the four hydroxybutyrates in saliva were easily quantified. Recently, Tsutsui et al. determined 3HB in human saliva by LC-P-ESI–MS/MS after derivatizing with (S)(+)-1-(2-pyrrolidinylmethyl)-pyrrolidine (Tsutsui et al. 2012). This method is also sensitive and enantiomeric separation of d- and l-3HB was achieved. However, 3HIB, 3HMB, and 2HB that are derived from amino acids were not quantified and 100 µL of saliva was used. Our method requires only 5 µL of saliva and can be applied to determination of the four hydroxybutyrates in lachrymal fluid collected with filter paper. Saliva and lachrymal fluid can be obtained non-invasively from humans and the concentrations of our four targeted hydroxybutyrates were significantly correlated among serum, saliva, and lachrymal fluid (data not shown). We chose saliva to monitor amino acids and lipid catabolic pathways because this can be more easily collected compared to lachrymal fluid. In addition, this high sensitive method can be applied to quantify the four hydroxybutyrates in other biological samples including serum, plasma, and urine that contain higher levels of them. Indeed, serum levels of 3HB, 3HIB, 3HMB, and 2HB were quantified in the present study (Fig. 8). In saliva, 3HB, 3HIB, and 3HMB concentrations were stabile at RT for at least 24 h (Fig. 7). However, 2HB concentration in saliva was decreased soon in several hours by standing at RT. The reason is unclear why salivary 2HB is unstable at RT, but saliva sample needs to freeze immediately after collection until analysis.

Our cell culture experiments confirmed that the four targeted hydroxybutyrates are metabolites of lipid and amino acids; i.e. 3HB was synthesized by hepatic fatty acid β-oxidation, 3HIB and 3HMB by skeletal muscular BCAA catabolism, and 2HB in the MET/THR/αKB pathways. In low nutritional states such as fasting, metabolic disorders and endurance exercise, 3HB serves as an excellent fuel for non-hepatic tissues and the serum level of 3HB is increased by stimulated hepatic β-oxidation as well as ketogenic amino acids catabolism (Laffel 1999). Elevation of serum 3HIB has also been found in fasted and diabetic subjects because BCAAs are utilized as another energy fuel in a low nutritional state (Avogaro and Bier 1989). The early steps in catabolism of the three BCAAs are thought to be concurrent reactions because the first two steps share the same enzymes: branched-chain aminotransferase and rate-limiting branched-chain α-keto acid dehydrogenase (BCKDH) complex (Shimomura et al. 2006). Therefore, the VAL metabolite 3HIB is thought to be a common biomarker for BCAA catabolism in skeletal muscle. Indeed, our data show that salivary levels of 3HB, 3HIB and 3HMB increased in parallel in patients with liver cirrhosis compared with those in healthy controls. Liver cirrhosis causes an imbalance of whole body energy metabolism, including impaired glucose tolerance, ketoacidosis and insulin resistance (Moriwaki et al. 2004). In skeletal muscles, BCAAs are used for compensatory energy production, and consequently protein breakdown and muscle atrophy develop in patients with liver cirrhosis (Shimomura et al. 2006; Platell et al. 2000; Moriwaki et al. 2004).

2HB is synthesized from αKB by a NADH-dependent 2-hydroxybutyrate dehydrogenase (2HBDH) in cytosol, and αKB is metabolized to propionyl-CoA and then to succinyl-CoA in mitochondria. Therefore, an elevated 2HB concentration appears to reflect increased activity of 2HBDH and/or decreased mitochondrial metabolism of αKB (Fig. 3). 2HBDH is an isozyme of lactate dehydrogenase (LDH), and production of 2HB from αKB is analogous to that of lactate from pyruvate (Landaas and Pettersen 1975). Metabolism of αKB to propionyl-CoA in mitochondria is thought to be catalyzed by a specific NAD^+^-dependent αKB dehydrogenase complex or other α-keto acid dehydrogenase complexes, including pyruvate dehydrogenase (PDH) and BCKDH (Linn et al. 1969b; Bremer 1969; Johnson and Connelly 1972). This activity is inhibited by phosphorylation of the E1 component of the complexes by enzymes such as pyruvate dehydrogenase kinase and BCKDH kinase (Kerbey et al. 1976). Gene expression of these kinases is upregulated by an elevated mitochondrial NADH/NAD ratio (Johnson and Connelly 1972; Linn et al. 1969a; Bremer 1969). Thus, elevation of this ratio increases the cellular αKB concentration through inhibition of αKB and α-keto acid dehydrogenase complexes, and an elevated cytosolic NADH/NAD ratio stimulates 2HB production through activation of 2HBDH.

αKB and pyruvate are structurally similar monocarboxylic keto acids and both are substrates of PDH; therefore, αKB may interfere with glucose metabolism by competitive inhibition of PDH (Lapointe and Olson 1985; Bremer 1969). αKB also inhibits transport of pyruvate into mitochondria (Paradies and Papa 1975). Brass showed that αKB significantly inhibited ^14^CO_2_ generation and [^14^C]glucose formation from [^14^C]pyruvate in rat hepatocytes, which implies that αKB reduces synthesis of acetyl-CoA and gluconeogenesis (Brass 1986). Early literature showed increased urinary and serum levels of 2HB in patients with various clinical disorders combined with ketoacidosis and lactic acidosis (Landaas and Pettersen 1975), but more recent studies suggest that serum 2HB is a significant biomarker associated with insulin sensitivity, diabetes mellitus and cardiovascular diseases (Syed Ikmal et al. 2013; Gall et al. 2010). In the current study, the salivary 2HB concentration in cirrhotic patients tended to be higher than that in healthy subjects. In cell culture, 2HB was produced by cultured hepatocytes when αKB, but not its precursors (MET, cystathionine or THR), was added. Therefore, increased 2HB levels in blood and saliva may reflect decreased mitochondrial metabolism of αKB, rather than increased catabolism of MET, cystathionine or THR. Serum 2HB might be an indicator of insulin resistance because accumulation of αKB results in elevation of 2HB and disturbance of mitochondrial pyruvate metabolism.

## Conclusion

In this study, four hydroxybutyrates (3HB, 3HIB, 3HMB, and 2HB) were converted to 2PM esters and measured by LC-P-ESI–MS/MS. This highly sensitive method made it possible to quantify concentrations of the four hydroxybutyrates in a small volume of human saliva, which can be conveniently used in place of serum. The four targeted hydroxybutyrates are intermediates in amino acid and fatty acid catabolic pathways that produce acetyl-CoA and succinyl-CoA in liver and skeletal muscles. Therefore, these four hydroxybutyrates are possible biomarkers for metabolic profiling of amino acids and lipids. This non-invasive method is applicable in clinical diagnosis, early detection of metabolic diseases, and evaluation of drug therapy; in nutritional support for patients and athletes; and in the anti-aging field.

## Additional files

**Additional file 1: Fig. S1.** Calibration curves for 3HB (**A**), 3HIB (**B**), 3HMB (**C**), and 2HB (**D**). Linearity was checked by simple linear regression. The equations for the best fit lines are (**A**) y = 0.0092x + 0.0020 (*n* = 8, r^2^ = 0.9998, *P* < 0.0001), (**B**) y = 0.0211x + 0.0032 (n = 7, r^2^ = 0.99998, *P* < 0.0001), (**C**) y = 0.0298x + 0.0043 (n = 6, r^2^ = 0.9991, *P* < 0.0001), and (**D**) y = 0.0322x + 0.0038 (*n* = 6, r^2^ = 0.9993, *P* < 0.0001). *Abbreviations:* *3HB*, 3-hydroxybutyrate; *3HIB*, 3-hydroxyisobutyrate; *3HMB*, 3-hydroxy-3-methylbutyrate; *2HB*, 2-hydroxybutyrate; *IS*, internal standard.
**Additional file 2: Table S1 and Table S2.** Reproducibility in the quantification of each hydroxybutyrate in human saliva.

## Abbreviations

2HB: 2-hydroxybutyrate
2HBDH: 2-hydroxybutyrate dehydrogenase
2PM: 2-pyridylmethyl
3HB: 3-hydroxybutyrate
3HBDH: 3HB dehydrogenase
3HIB: 3-hydroxyisobutyrate
3HIB-CoA: 3-hydroxyisobutyryl-CoA
3HMB: 3-hydroxy-3-methylbutyrate
αKADH: α-keto acid dehydrogenase
αKB: α-ketobutyrate
α-Kg: α-ketoglutaric acid
Ac-CoA: acetyl-CoA
AcetoAc-CoA: acetoacetyl-CoA
BCAA: branched-chain amino acid
BCAT: branched-chain aminotransferase
BCKA: branched-chain α-keto acid
BCKDH complex: branched-chain α-keto acid dehydrogenase complex
CTH: γ-cystathionase
CYS: cysteine
FFA: fatty acids
GC–MS: gas chromatography–mass spectrometry
GLU: glutamate
GSH: glutathione
HCY: homocysteine
Inner m.: mitochondrial inner membrane
KIC: α-ketoisocaproic acid
KICDO: KIC dioxygenase
KMV: α-keto-β-methylvaleric acid
KIV: α-ketoisovaleric acid
HIBCH: 3-hydroxyisobutyryl-CoA hydrolase
HMG-CoA: 3-hydroxy-3-methylglutaryl-CoA
HMGCL: HMG-CoA lyase
HMGCS: HMG-CoA synthase
IB-CoA: isobutyryl-CoA
ILE: isoleucine
Inner m.: mitochondria inner membrane
IS: internal standard
IV-CoA: isovaleryl-CoA
LC–MS/MS: liquid chromatography–tandem mass spectrometry
LDH: lactate dehydrogenase
LEU: leucine
LYS: lysine
MET: methionine
MB-CoA: α-methylbutyryl-CoA
MC-CoA: methacrylyl-CoA
MMS: methylmalonate semialdehyde
N-ESI: negative electrospray ionization
Outer m.: mitochondrial outer membrane
Palmitic A: palmitic acid
PDH: pyruvate dehydrogenase
P-ESI: positive electrospray ionization
Plasma m.: plasma membrane
PPARα: peroxisome proliferator-activated receptor α
PP-CoA: propionyl-CoA
RT: room temperature
SAM: *S*-adenosyl methionine
SAH: *S*-adenosyl homocysteine
SER: serine
SDH: serine/threonine dehydrase
S/N: signal-to-noise ratio
SRM: selected reaction monitoring
Suc-CoA: succinyl-CoA
THR: threonine
TRP: tryptophan
TYR: tyrosine
UFLC: ultra-fast liquid chromatography
VAL: valine
