# Supplementary figures and images for: Simultaneous quantification of salivary 3-hydroxybutyrate, 3-hydroxyisobutyrate, 3-hydroxy-3-methylbutyrate, and 2-hydroxybutyrate as possible markers of amino acid and fatty acid catabolic pathways by LC–ESI–MS/MS
Source: Springerplus. 2015 Sep 15;4:494. doi: 10.1186/s40064-015-1304-0 (PMC4571036; doi:10.1186/s40064-015-1304-0)

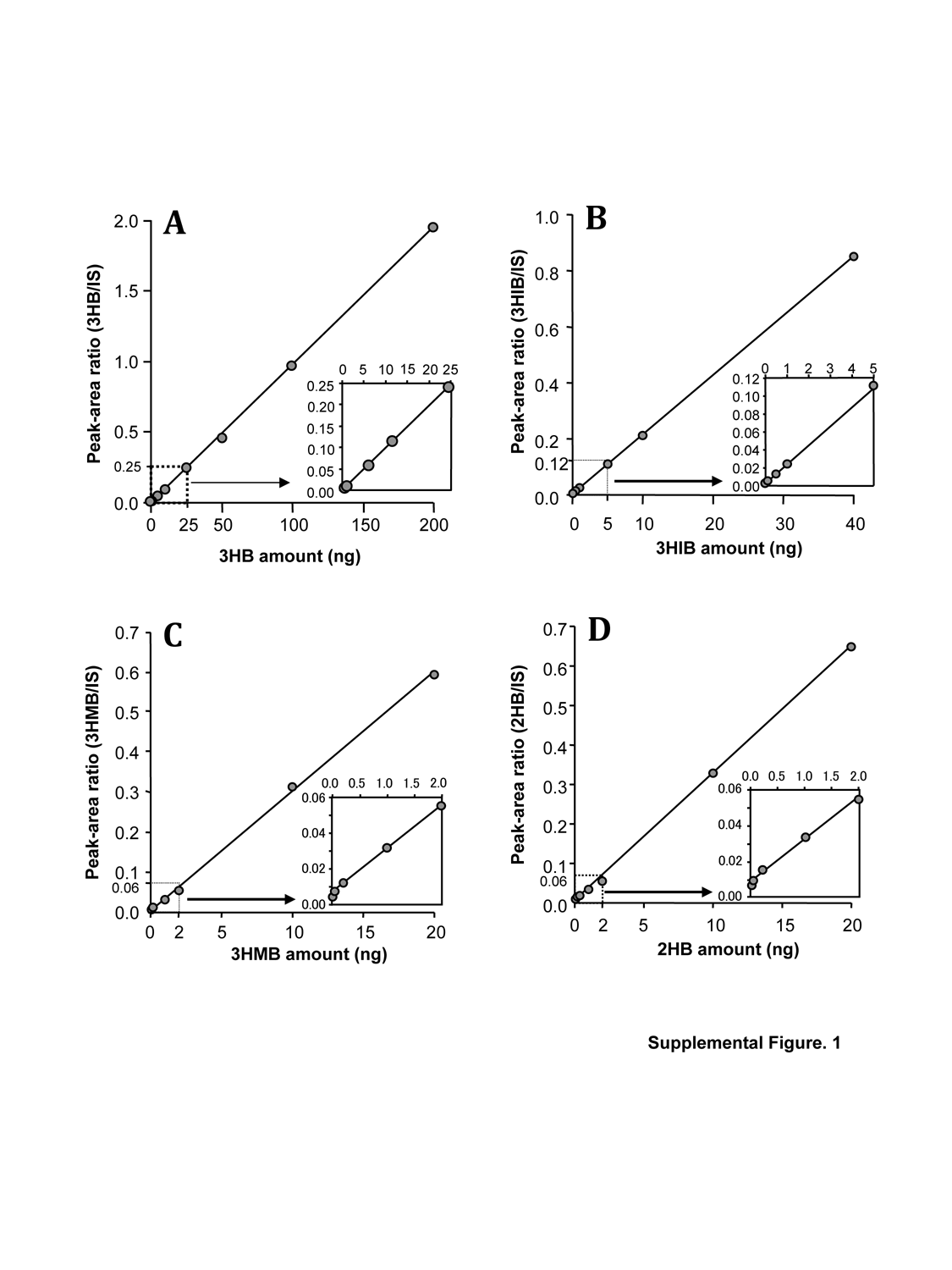

Supplement: Supplementary file 1 — Additional file 1: Fig. S1. Calibration curves for 3HB (A), 3HIB (B), 3HMB (C), and 2HB (D). Linearity was checked by simple linear regression. The equations for the best fit lines are (A) y = 0.0092x + 0.0020 (n = 8, r2 = 0.9998, P < 0.0001), (B) y = 0.0211x + 0.0032 (n = 7, r2 = 0.99998, P < 0.0001), (C) y = 0.0298x + 0.0043 (n = 6, r2 = 0.9991, P < 0.0001), and (D) y = 0.0322x + 0.0038 (n = 6, r2 = 0.9993, P < 0.0001). Abbreviations: 3HB, 3-hydroxybutyrate; 3HIB, 3-hydroxyisobutyrate; 3HMB, 3-hydroxy-3-methylbutyrate; 2HB, 2-hydroxybutyrate; IS, internal standard. [file 40064_2015_1304_MOESM1_ESM.tif]
