# Supplementary material for: Simultaneous quantification of salivary 3-hydroxybutyrate, 3-hydroxyisobutyrate, 3-hydroxy-3-methylbutyrate, and 2-hydroxybutyrate as possible markers of amino acid and fatty acid catabolic pathways by LC–ESI–MS/MS
Source: Springerplus. 2015 Sep 15;4:494. doi: 10.1186/s40064-015-1304-0 (PMC4571036; doi:10.1186/s40064-015-1304-0)
Supplement: Supplementary file 2 — Additional file 2: Table S1 and Table S2. Reproducibility in the quantification of each hydroxybutyrate in human saliva. [file 40064_2015_1304_MOESM2_ESM.doc]

| **Supplemental Table S1.** Reproducibility in the quantification of each hydroxybutyrate in human saliva: analytical data | | | | | | |
| --- | --- | --- | --- | --- | --- | --- |
| Sample | | Individual Values | | |  | Mean ± SD |
| ***3HB*** | | *pmol* | | | | |
| A | | 56.5 | 56.0 | 56.4 |  | 56.3 ± 0.2 |
| B | | 55.9 | 56.2 | 55.9 |  | 56.0 ± 0.2 |
| C | | 55.7 | 55.7 | 56.3 |  | 55.9 ± 0.4 |
| D | | 56.0 | 55.6 | 55.8 |  | 55.8 ± 0.2 |
| Mean ± SD | |  |  |  |  | 56.0± 0.3 |
|  | |  |  |  |  |  |
| ***3HIB*** | | *pmol* | | | | |
| A | | 49.2 | 48.4 | 49.2 |  | 48.9 ± 0.5 |
| B | | 49.2 | 49.2 | 49.2 |  | 49.2 ± 0.0 |
| C | | 49.2 | 48.4 | 49.2 |  | 48.9 ± 0.5 |
| D | | 49.2 | 49.2 | 48.4 |  | 48.9 ± 0.5 |
| Mean ± SD | |  |  |  |  | 49.0 ± 0.4 |
|  | |  |  |  |  |  |
| ***3HMB*** | | *pmol* | | | | |
| A | | 3.8 | 3.6 | 3.8 |  | 3.8 ± 0.1 |
| B | | 3.8 | 3.6 | 3.8 |  | 3.7 ± 0.1 |
| C | | 3.6 | 3.8 | 3.8 |  | 3.7 ± 0.2 |
| D | | 3.7 | 3.7 | 3.6 |  | 3.7 ± 0.1 |
| Mean ± SD | |  |  |  |  | 3.7 ± 0.1 |
|  | |  |  |  |  |  |
| ***2HB*** | | *pmol* | | | | |
| A | | 19.9 | 20.0 | 19.8 |  | 19.9 ± 0.1 |
| B | | 20.7 | 20.2 | 20.2 |  | 20.3 ± 0.3 |
| C | | 18.3 | 18.3 | 20.1 |  | 18.9± 1.1 |
| D | | 20.1 | 18.2 | 20.0 |  | 19.4 ± 1.0 |
| Mean ± SD | |  |  |  |  | 19.5 ± 0.8 |
|  | Data are shown as mean ± SD in triplicate experiment. Each hydroxybutyrate was quantified in 5 µL of normal human saliva. *3HB*, 3-hydroxybutyrate; *3HIB*, 3-hydroxyisobutyrate; *3HMB*, 3-hydroxy-3-methylbutyrate; *2HB*, 2-hydroxybutyrate | | | | | |

| **Supplemental Table S2.** Reproducibility in the quantification of hydroxybutyrates in human saliva: ANOVA | | | | | | |
| --- | --- | --- | --- | --- | --- | --- |
| Source | | *S* | *F* | *V* | *F_0_* | Relative SD |
|  | |  |  |  |  |  |
| ***3HB*** | |  |  |  |  | % |
| Sample preparation | | 0.452 | 3 | 0.150 | 2.19 | 0.45 |
| Error (SRM) | | 0.550 | 8 | 0.068 |  | 0.54 |
| Total | | 1.001 | 11 |  |  |  |
|  | |  |  | *F(3,8,0.05)* = 4.07 | |  |
| ***3HIB*** | |  |  |  |  | % |
| Sample preparation | | 0.157 | 3 | 0.052 | 0.33 | 0.45 |
| Error (SRM) | | 1.259 | 8 | 0.157 |  | 0.79 |
| Total | | 1.417 | 11 |  |  |  |
|  | |  |  | *F(3,8,0.05)* = 4.07 | |  |
|  | |  |  |  |  |  |
| ***3HMB*** | |  |  |  |  | % |
| Sample preparation | | 0.014 | 3 | 0.0047 | 0.39 | 1.70 |
| Error (SRM) | | 0.098 | 8 | 0.0123 |  | 2.98 |
| Total | | 0.112 | 11 |  |  |  |
|  | |  |  | *F(3,8,0.05)* = 4.07 | |  |
|  | |  |  |  |  |  |
| ***2HB*** | |  |  |  |  | % |
| Sample preparation | | 3.22 | 3 | 1.076 | 1.90 | 5.28 |
| Error (SRM) | | 4.53 | 8 | 0.567 |  | 3.45 |
| Total | | 7.76 | 11 |  |  |  |
|  | |  |  | *F(3,8,0.05)* = 4.07 | |  |
|  | *S*, residual sum of squares; *f*, number of degrees of freedom; *f_1_*, *f_sample preparation_*; *f2*, *f_error_*; *V*, unbiased variance; *F_0_*, observed value following *F* distribution variance ratio (*V_sample_ _preparation_*/*V_error_*); *F*(*f_1_*, *f_2_*, *α*), density function of *F* distribution with *f_1_* and *f_2_* degree of freedom, *3HB*, 3-hydroxybutyrate; *3HIB*, 3-hydroxyisobutyrate; *3HMB*, 3-hydroxy-3-methylbutyrate; *2HB*, 2-hydroxybutyrate. | | | | | |

| **Supplemental Table 3.** Recovery of the hydroxybutyrates from human saliva | | | | | | | |
| --- | --- | --- | --- | --- | --- | --- | --- |
| Sample (*X_0_* + na)  (n = 0,1,2,3) | | Amount Added | Amount Found | | | Recovery *^b^*  (Mean ± SD) | Estimated Amount ± 95% Confidence Limit *^c^* |
| ***3HB*** | |  | *pmol* | | | % | *pmol* |
| *X_0_* | | 0.0 | *X_0_* ± SD = 56.0± 0.30 *^a^* | | |  | 58.1 ± 2.6 |
| *X_0_*+a | | 51.2 | 111.5 | 111.7 | 110.9 |  |  |
| *X_0_*+a | | 51.2 | 107.8 | 108.0 | 108.2 | 103.4 ± 3.6 |  |
| *X_0_*+2a | | 103.2 | 165.2 | 167.1 | 163.2 |  |  |
| *X_0_*+2a | | 103.2 | 163.9 | 163.5 | 162.5 | 104.6 ± 1.6 |  |
| *X_0_*+3a | | 154.4 | 214.6 | 211.3 | 214.0 |  |  |
| *X_0_*+3a | | 154.4 | 216.0 | 214.5 | 215.5 | 102.1 ± 1.1 |  |
|  | |  |  | | |  |  |
| ***3HIB*** | |  | *pmol* | | | % | *pmol* |
| *X_0_* | | 0.0 | *X_0_* ± SD = 49.0 ± 0.36 *^a^* | | |  | 47.4 ± 1.6 |
| *X_0_*+a | | 50.0 | 100.3 | 98.6 | 98.1 |  |  |
| *X_0_*+a | | 50.0 | 98.4 | 97.7 | 97.6 | 98.5 ± 2.0 |  |
| *X_0_*+2a | | 100.0 | 147.8 | 149.4 | 148.5 |  |  |
| *X_0_*+2a | | 100.0 | 149.3 | 150.3 | 147.9 | 99.7 ± 1.0 |  |
| *X_0_*+3a | | 150.0 | 201.5 | 202.0 | 201.6 |  |  |
| *X_0_*+3a | | 150.0 | 199.1 | 198.2 | 199.4 | 100.7 ± 1.1 |  |
|  | |  |  |  |  |  |  |
| ***3HMB*** | |  | *pmol* | | | % | *pmol* |
| *X_0_* | | 0.0 |  *X_0_* ± SD = 3.7 ± 0.10 *^a^* | | |  | 3.6 ± 0.1 |
| *X_0_*+a | | 3.8 | 7.3 | 7.3 | 7.2 |  |  |
| *X_0_*+a | | 3.8 | 7.3 | 7.3 | 7.3 | 104.8 ± 1.5 |  |
| *X_0_*+2a | | 7.6 | 11.1 | 11.1 | 11.0 |  |  |
| *X_0_*+2a | | 7.6 | 11.0 | 11.0 | 11.0 | 107.7 ± 0.6 |  |
| *X_0_*+3a | | 11.4 | 14.7 | 14.6 | 14.6 |  |  |
| *X_0_*+3a | | 11.4 | 14.7 | 14.8 | 14.6 | 107.9 ± 0.9 |  |
|  | |  |  |  |  |  |  |
| ***2HB*** | |  | *pmol* | | | % | *pmol* |
| *X_0_* | | 0.0 |  *X_0_* ± SD = 19.5 ± 0.84 *^a^* | | |  | 19.6 ± 1.4 |
| *X_0_*+a | | 19.2 | 41.6 | 40.2 | 40.8 |  |  |
| *X_0_*+a | | 19.2 | 38.9 | 41.7 | 40.0 | 108.8 ± 5.4 |  |
| *X_0_*+2a | | 38.4 | 62.8 | 60.7 | 61.6 |  |  |
| *X_0_*+2a | | 38.4 | 61.9 | 60.8 | 60.9 | 108.8 ± 2.2 |  |
| *X_0_*+3a | | 57.6 | 83.9 | 82.9 | 81.7 |  |  |
| *X_0_*+3a | | 57.6 | 81.7 | 80.0 | 83.7 | 108.7 ± 2.5 |  |
|  | Known amounts of each butyrates were spiked into 5 µL of normal human saliva before sample preparation. *a* The value was obtained from Supplemental Table 1. *b* Recovery (%) = (amount found - *X_0_*) / amount added × 100. *c* The estimated amount was calculated by orthogonal regression. *3HIB*, 3-hydroxyisobutyrate; *3HMB*, 3-hydroxy-3-methyl-butyrate; *2HB*, 2-hydroxybutyrate. | | | | | | |
